# 5-year efficacy of all surface laser ablation with cross-linking (ASLA-XTRA) for the treatment of myopia

**DOI:** 10.1186/s40662-020-00198-z

**Published:** 2020-06-11

**Authors:** Ioannis M. Aslanides, Farhad Hafezi, Shihao Chen, Hatch Mukherjee, Vasileios Selimis, Ilias Maragkos, Nanji Lu, George Kymionis

**Affiliations:** 1 Emmetropia Mediterranean Eye Institute, Plateia Eleftherias 44, Heraklion, 71201 Crete, Greece; 2 Eye Hospital, Wenzhou Medical University, Wenzhou, China; 3 Center for Applied Biotechnology and Molecular Medicine, University of Zurich, Dietikon/Zurich, Switzerland; 4 ELZA Institute, Dietikon/Zurich, Switzerland; 5 USC Roski Eye Institute, University of Southern California, California, Los Angeles USA; 6 Faculty of Medicine, University of Geneva, Geneva, Switzerland; 7 Essex County Hospital, Essex, UK; 8 Athens Medical School, University of Athens, Athens, Greece; 9 Faculty of Biology and Medicine, Jules Gonin Eye Hospital, University of Lausanne, Lausanne, Switzerland

**Keywords:** Refractive surgery, Transepithelial, PRK, ASLA, Cross-linking, CXL, ASLA-XTRA

## Abstract

**Background:**

The purpose of our study is to examine the long (5-year) efficacy of the all surface laser ablation (ASLA) combined with accelerated cross-linking (CXL) for the treatment of myopia without the use of mitomycin-C (MMC).

**Methods:**

This retrospective study consisted of 202 eyes of 118 myopic (SD: 2.41, range: − 1.50 to − 12.75 D) patients (44 males, 74 females). Mean age was 28.50 years (SD: 6.45, range: 18 to 51 years) that underwent ASLA with accelerated CXL for the treatment of their myopia.

**Results:**

The patients underwent routine postoperative assessment on the 1st, 3rd, 7th day and in the 1st, 3rd, 6th and 12th month, 30th month (±6 months), 4th and 5th year. The mean spherical equivalent (SEq) refractive error changed from − 6.41 ± 2.41 D preoperatively to − 0.02 ± 0.53 D at 5 years postoperatively. The haze score was 0.18, 0.25 and 0.28 at 1, 3 and 6 months postoperatively. At 12 months after the treatment, no eyes had significant corneal haze and in all the following postoperative time intervals the haze traces were gone.

**Conclusion:**

ASLA combined with accelerated CXL (ASLA-XTRA) appears to be safe, efficacious and offering very good refractive results. The potential additional benefits of this modality are the stabilizing effect of the refraction and its sterilization effect on the treated cornea without the potential side effects of MMC.

## Introduction

Photorefractive keratectomy (PRK) is a well-known procedure for the correction of myopia by ablating the surface of the cornea after removing the epithelium by mechanical or chemical debridement. The primary issue following surface ablation methods is the incidence of postoperative corneal haze because of the wound healing process after the treatment, as it may lead to a substantial decrease of visual acuity and may possibly necessitate further interventions in order to enhance the patient’s vision. However, the introduction of mitomycin-C (MMC) was a significant step in the re-evaluation of surface ablation techniques as it has been proven to have a positive effect on the prevention of postoperative corneal haze formation [1–3].

A similar method named tPRK (PTK-PRK) consists of initial laser epithelial removal with PTK – usually using a flat rather than refractive neutral profile – followed by a PRK treatment at a second distinct step, is also well described [4–11].

However, in recent years, a modified excimer laser trans-epithelial surface ablation technique was introduced by Schwind eye-tech-solutions, Germany) [7, 10, 12, 13]. This technique, although different in mechanism, was named also, inappropriately in our opinion, Trans-PRK. More specifically, in this technique, the whole treatment is performed in one step combining both epithelium and stromal ablation in a single-step ablation profile. In this modality, unlike tPRK, the epithelium is removed by the excimer laser based on customized epithelial thickness profile [14–16] in a way that assures refractive neutrality. The other difference is that the refractive component of the treatment profile is initially applied on the cornea followed by the epithelial one [7]. Therefore, in order to avoid any kind of confusion when referring to Schwind’s Trans-PRK, we have introduced and been using the term all surface laser ablation (ASLA) in all our previous studies [7, 17–19].

The well-established PTK and PRK treatments have also both been studied before in combination with corneal cross-linking (CXL) procedures in order to improve stability and reduce the refractive error in cases of ectasia, such as keratoconus. In this setting, CXL acted as a stabilizing intervention on the degenerated cornea [19–21]. It has been demonstrated [22, 23] that, after the CXL treatment, once the epithelial defect has fully healed, there is transformation and then apoptosis of the myofibroblasts, followed by subsequent repopulation by new keratocytes, and reabsorption of the myofibroblast derived abnormal collagen depositions and cell debris, this process enhancing corneal clarity.

We hypothesized that this cellular process induced by CXL may have a beneficial effect on reducing subepithelial haze following ASLA transepithelial PRK. The aim of this research study was to investigate the 5-year efficacy, the effectiveness in preventing haze formation and the safety of the previously described ASLA-XTRA [18] modality which consists of ASLA followed by accelerated CXL for the treatment of patients with moderate to high myopia avoiding MMC and its potential side effects (e.g., corneal melting, endothelial cell loss) [24].

## Materials and methods

This study was performed at Emmetropia Eye Institute in Greece. To be included in this retrospective study, all patients had to be 18 years old or more with myopic spherical equivalent (SEq) refraction stable for more than 1 year. Patients with ocular co-pathology or previous surgery, inflammatory or infectious corneal disease, systemic diseases (with ocular involvement) and keratoconus were excluded. Three weeks prior to surgery, patients discontinued wearing contact lenses. All participants were informed before their participation in the study as well as the risks and the advantages of the surgery and signed a written paper before the treatment. The study was conducted following the principles of the Declaration of Helsinki and ethical approval was provided by the Emmetropia’s institutional review board.

### Preoperative assessment

The preoperative assessment included uncorrected (UDVA) and corrected (CDVA) distant visual acuity, non-cycloplegic and cycloplegic manifest refraction, and slit lamp examination, including dilated fundoscopy and measurement of intraocular pressure (ICare, ic100, ICare Finland, Oy, Finland). The preoperative assessment also included corneal topography using Orbscan IIz (Bausch and Lomb, Rochester, NY, USA). Pachymetric and epithelial thickness measurements were performed using Optovue OCT (Optovue, Frement, CA, USA).

### Surgical technique

The treatments were performed using the Schwind AMARIS 750S excimer laser platform and had aberration-free (aspheric) profiles.

Patients were given 1.5 mg bromazepam (Lexotanil, Roche Hellas Α.Ε.) preoperatively and one drop of proparacaine (Alcaine, Alcon, USA) was instilled three times 15 min before surgery. Povidone iodine 5% solution (Betadine) was applied on the eyes prior to the application of an upper lid drape and of a speculum. Wet sponge application (three slow, painting movements on the corneal surface) was performed using a Merocel® sponge (Medtronic Inc., Minneapolis, MN, USA) dipped in balanced salt solution (BSS) in order to avoid uneven wetting and, therefore, uneven ablation. After the ASLA treatment was performed, the cornea was chilled with 20 mL of low temperature BSS (4 °C). Following ASLA, an accelerated CXL treatment was performed using the Avedro KXL system (Avedro Inc., Waltham, MA). The riboflavin solution used was Vibex Rapid (Avedro Inc., Waltham, MA), the formulation of which is: 0.1% riboflavin, Saline, HPMC. The soaking time before the UV irradiation was 90s and the energy used 2.7 J/cm^2^ (30 mW for 90 s). Finally, a drop of Ofloxacin 0.3% (Exocin, Allergan Inc., Irvine, CA, USA) was applied and a bandage contact lens (Acuvue Oasys; Johnson & Johnson, Jacksonville, FL, USA). After surgery, topical Ofloxacin 0.3% (Exocin, Allergan Inc., Irvine, CA, USA) was applied four times per day until complete epithelial closure and removal of the contact lens. Dexamethasone 0.1% eye drops (Maxidex, Novartis, Basel, Switzerland) qid were used for 1–2 weeks, replaced with fluorometholone alcohol 0.1% (FML, Allergan Inc., Irvine, CA, USA) for 10 weeks, and artificial tears for 3 months (Thealoz Duo Eye Drops; Laboratoires Thea, France) were prescribed.

### Treatment parameters

The average maximum ablation depth was 150.65 μm (SD: 29.96, range 81.17 to 226.78 μm). This also includes the epithelium according to the epithelial profile used by Schwind (55 μm centrally). As a result, the average stromal ablation depth was 95.65 μm (range 26.17 to 171.78 μm) while the residual stromal thickness was 385.56 μm (SD: 40.27, range 316.26 to 500.39 μm). The mean optical zone was 6.29 mm (SD: 0.26, range 5.7 to 7 mm). In all cases, the choice of the optical zone was made following the manufacturer’s guidelines taking also into consideration the amount of the tissue what would be ablated in each case, the pupil size and the pachymetric values of each patient. As a result, the optical zone was reduced, compared to the manufacturer’s suggestions, in some cases. The CXL energy used was 2.7 J/cm^2^. There were no intraoperative complications during the treatments.

### Postoperative follow up

Patients underwent routine postoperative assessment on the 1st, 3rd, 7th day and in the 1st, 3rd, 6th and 12th month, 30th month (±6 months), 4th and 5th year. During the postoperative period from 1 month onwards, subjective refraction, slit lamp biomicroscopy, intraocular pressure measurement were performed. The corneal haze was graded with an ordinal scale described by Fantes et al. [25]

## Results

Two hundred and two eyes (99 right and 103 left eyes) of 118 patients meeting the inclusion criteria were included. There were 44 male and 74 female patients. Mean age was 28.5 years (SD: 6.45, range 18 to 51). The mean pachymetry value and Kmax values before the treatment were 536.21 μm (SD: 42.80, range 434 to 650) and 45.06 D (SD: 1.71, range 40.5 to 50.1), respectively. The epithelium had a mean value of 54.94 μm (SD: 1.04, range 52 to 58). Mean preoperative SEq refractive error was − 6.41 D (SD: 2.41, range − 1.5 to − 12.75).

Mean pre-operative CDVA was 0.018 logMAR (SD: 0.069, range 0.3 to − 0.2) and the mean pre-operative UDVA was 2.745 logMAR (SD: 0.68, range 3 to 0.54).

At 12 months post-operatively (113 eyes), the SEq was − 0.08 D (SD: 0.36, range − 1.50 to 0.625). Mean CDVA at 12 months was 0.008 logMAR (SD: 0.69 range 0.3 to − 0.1) and the mean UDVA at the same period was 0.03 logMAR (SD: 0.09, range 0.4 to − 0.1).

At 30 ± 6 months post-operatively (90 eyes), the SEq was − 0.23 D (SD: 0.43, range − 1.50 to 0). Mean CDVA at 30 ± 6 months was 0.008 logMAR (SD: 0.05 range 0.18 to − 0.1) and the mean UDVA at the same period was 0.04 logMAR (SD: 0.07, range 0.18 to − 0.1).

At 4 years post-operatively (83 eyes), the SEq was − 0.14 D (SD: 0.38, range − 1.375 to 0.25). Mean CDVA at 4 years was 0.003 logMAR (SD: 0.04 range 0.2 to − 0.1) and the mean UDVA at the same period was 0.05 logMAR (SD: 0.1, range 0.3 to − 0.1).

**Fig. 1:**
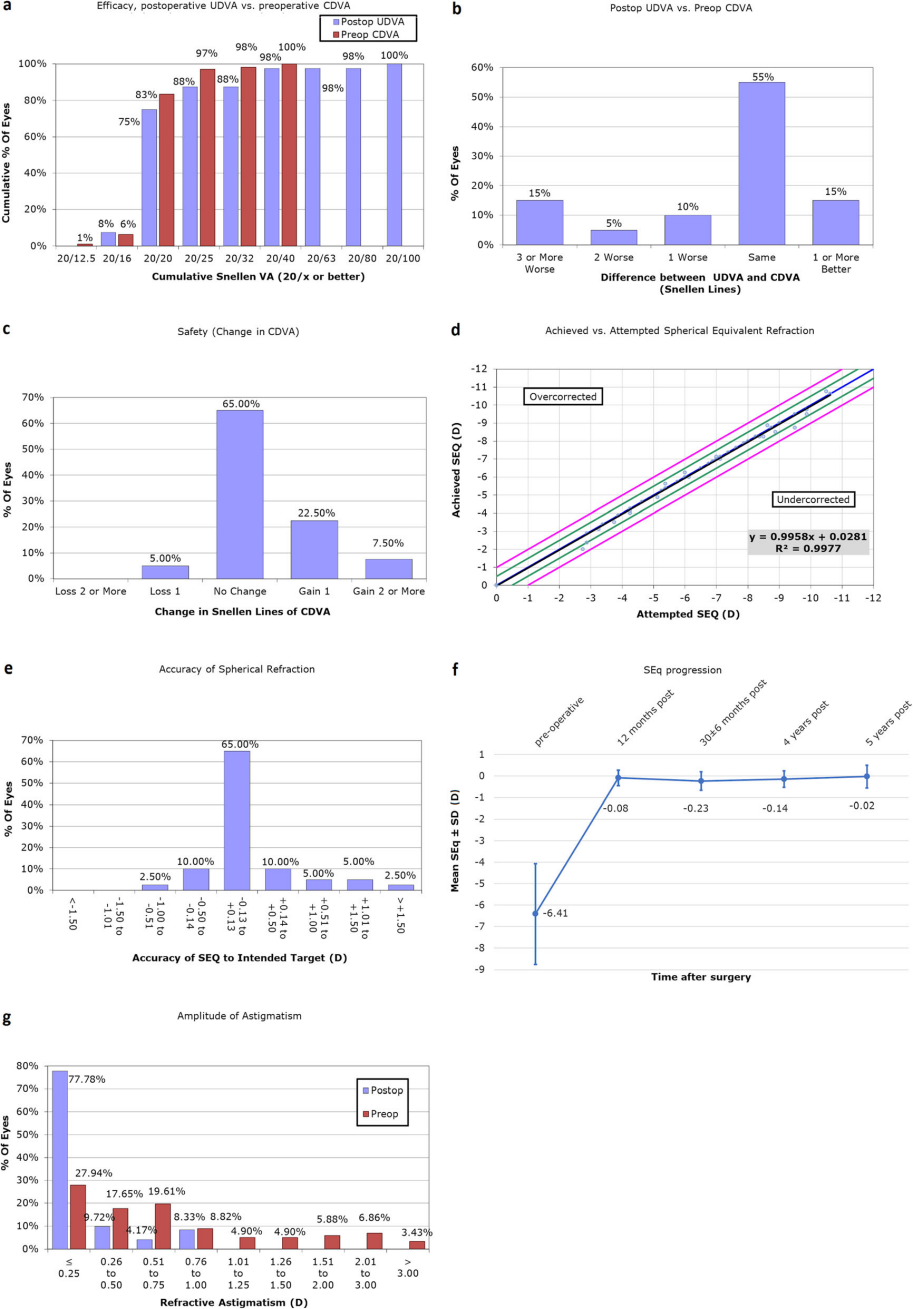
ASLA-XTRA long term results regarding the **a**) Efficacy of the method (postoperative UDVA vs. preoperative CDVA, **b**) Difference between the UDVA and the CDVA (in Snellen Lines), **c**) Safety (change in CDVA), **d**) Achieved vs. attempted spherical equivalent refraction, **e**) Accuracy of the spherical equivalent refraction, **f**) Stability of the spherical equivalent refraction and **g**) Amplitude of the astigmatism

At 5 years post-operatively (72 eyes), the SEq was − 0.02 D (SD: 0.53, range − 1.625 to 1.5). Mean CDVA at 5 years was 0.007 logMAR (SD: 0.05 range 0.2 to − 0.1) and the mean UDVA at the same period was 0.064 logMAR (SD 0.18, range 1 to − 0.1) (Fig. 1).

**Fig. 2:**
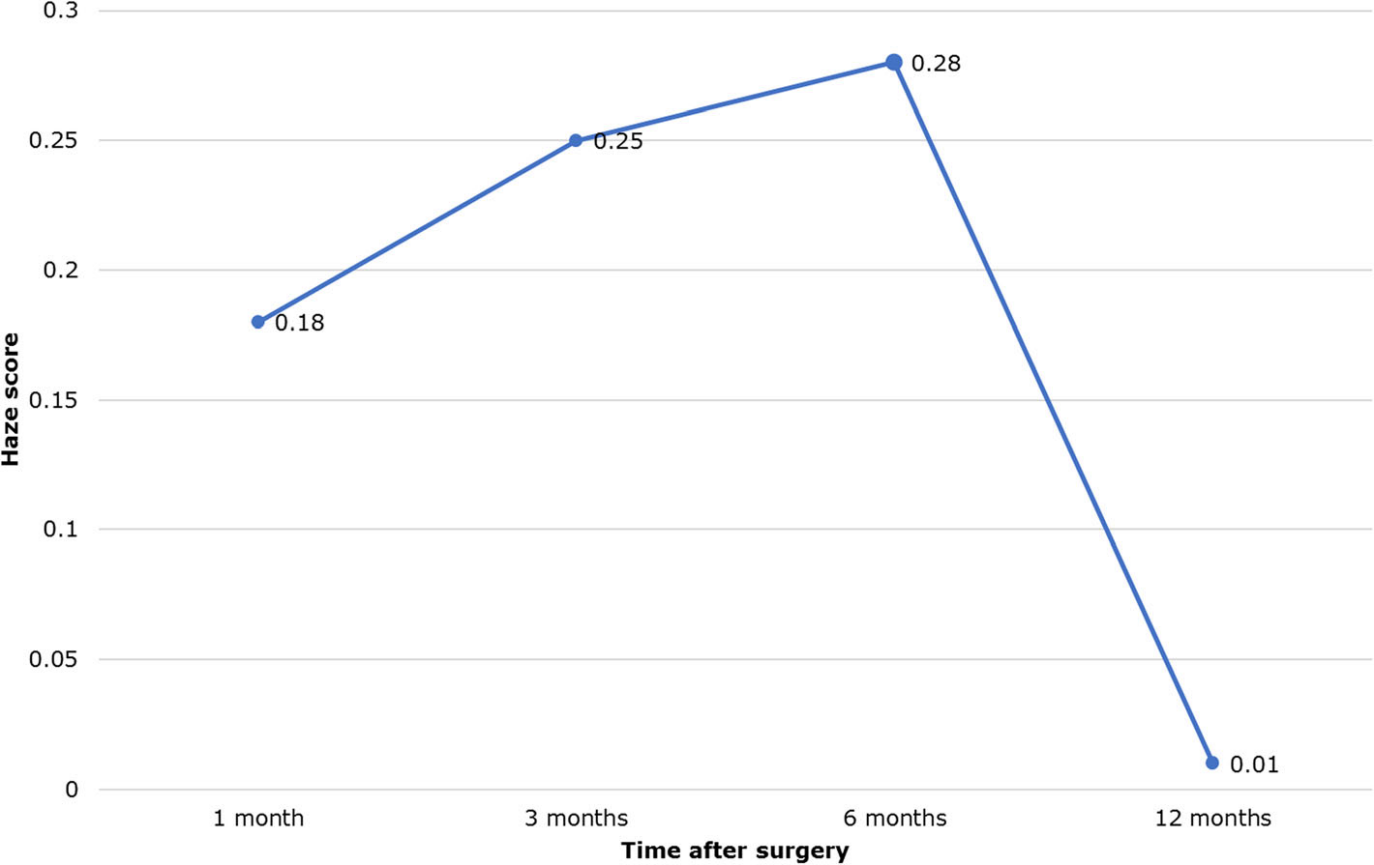
Haze score postoperatively

Regarding haze, the haze score was 0.18, 0.25 and 0.28 at 1, 3 and 6 months postoperatively. At 12 months after the treatment, no eyes had significant corneal haze (only insignificant haze traces were present in a few eyes). In all the following postoperative time intervals the haze traces were gone (Fig. 2).

## Discussion

In this study we elaborate on our recent publication [18], a comparative study suggesting the use of accelerated CXL instead of MMC for the prevention of postoperative corneal haze formation, by reporting the long term outcomes of ASLA-XTRA. We used a total energy of 2.7 J/cm^2^ which is half the energy delivered during conventional CXL following the Dresden protocol. The main goal of this accelerated, superficial fluence is to maintain the corneal strength without inducing a refractive change. The results of the current study seem to corroborate the results of the original study comparing ASLA with MMC vs. ASLA-XTRA. The refractive outcome is stable 5 years after the treatment with good results in terms of UDVA, there was no loss of 2 or more lines of BCVA in any case, and the postoperative refraction that are very similar to the existing literature. There were ~ 3–4 eyes (patients older than 50 years old) that lost one line of BCVA but that loss has occurred merely due to lenticular changes and not because of corneal pathology.

According to our long-term dataset, the ASLA-XTRA technique seems to have very encouraging results in terms of preventing corneal haze formation while maintaining good efficacy and stability. The only haze formation that was observed was from the 1st and until the 6th month postoperatively and we speculate that it was caused also by the CXL procedure itself and, in any case, did not affect the patients’ vision significantly. Lastly, randomized blind comparative studies of MMC vs. CXL, with larger sample size, concerning the safety, the efficacy and the prevention of haze after PRK, are needed in the future to corroborate our results.

## Conclusions

We favor the use of CXL in place of MMC in refractive laser ablation mainly because we believe that CXL has similar MMC-like effect on fibroblast activity in decreasing haze formation. In addition, it contributes to the postoperative biomechanical stability of the cornea and, as a result, a refraction that could potentially be more stable in the long term. Finally, CXL offers a cornea sterilizing effect, thanks to the combination of riboflavin and UV radiation, and consequently may reduce the possibility of postoperative infections.

Overall, our long-term data have comparable incidence of haze to published incidence with the use of MMC [2, 17, 18, 26]. This suggests that both the topical application of MMC 0.02% and accelerated CXL, by having the same effect on the corneal fibroblasts, are equally effective in preventing the long-term development of post-operative corneal haze, with equivalent refractive results, in moderate to high myopic patients. In this regard it is worth noting that the deep haze seen after conventional CXL differs from the subepithelial haze after PRK, with the latter being the target of this intervention. In this regard, it is also noteworthy that unlike other studies suggesting visually significant haze following combined excimer ablation/ CXL, our protocol did not use a full 30 min CXL treatment, but rather a highly accelerated and reduced energy treatment, which may explain the lack of deep stromal or visually significant haze in our study [27]. Overall, the combination of stability, lack of haze and sterilizing effect make ASLA-XTRA our favorite methodology even though compared to MMC, the CXL treatment is less cost-effective.

## Data Availability

Data supporting the results reported in the article are not public but can be accessed after communicating with the corresponding author.
